# Effects of geopolitical risk on environmental sustainability and the moderating role of environmental policy stringency

**DOI:** 10.1038/s41598-024-60773-5

**Published:** 2024-05-10

**Authors:** Heng Luo, Ying Sun

**Affiliations:** 1 School of Digital Economy and Industry, Jiangxi University of Engineering, Xinyu, Jiangxi China; 2 School of Business and Economics, Universiti Putra Malaysia, Serdang, Malaysia

**Keywords:** Geopolitical risk, Consumption-based CO_2_ emissions, Environmental policy stringency, Quantile regression, Environmental economics, Environmental impact

## Abstract

This study investigates the impact of geopolitical risk (GPR) on consumption-based carbon (CCO_2_) emissions as well as the moderating role of environmental policy stringency (EPS) on the above relationship. Based on data collected from 27 countries from 1990 to 2020, the basic results from the sample of the study indicate that GPR accelerates CCO_2_ emissions. Quantile regression results reveal that the effect of GPR is more pronounced in countries with higher CCO_2_ emissions. Moreover, EPS weakens the escalating effect of GPR on CCO_2_ emissions. The robust test results validate the findings reported in the basic regression model. The heterogeneity test indicates that the impact of GPR on CCO_2_ emissions is greater in developing countries compared in developed countries. The study also proposes these policy implications based on the findings: (1) countries should ensure a stable political environment, establish a robust legal system and promote energy transition; and (2) the scope of environmental taxes should be expanded where different tax rates should be imposed in order to be useful in reducing CCO_2_ emissions.

## Introduction

Environmental sustainability has become a prominent issue as it is essential to both economic progress and human health. This has led to alliances among nations and international institutions to adopt efficient measures that are driven by concerns on environmental deterioration. With the goal of reducing greenhouse gas emissions and promoting environmental sustainability, nations from around the world have taken part in conferences like the Conference of Parties (COP) series as well as international agreements like the Kyoto Protocol, the Paris Agreement, and the United Nations Framework Convention on Climate Change. The COP 26, for instance, set a global goal to reduce existing carbon dioxide emissions by 50% of 2010 levels^1^. Following that, COP 27 underscored the significance of climate change and the necessity for global cooperation to attain carbon neutrality. In addition, COP 28 introduced the global stocktake and pledges to transition away from fossil fuels in energy systems.

**Figure 1:**
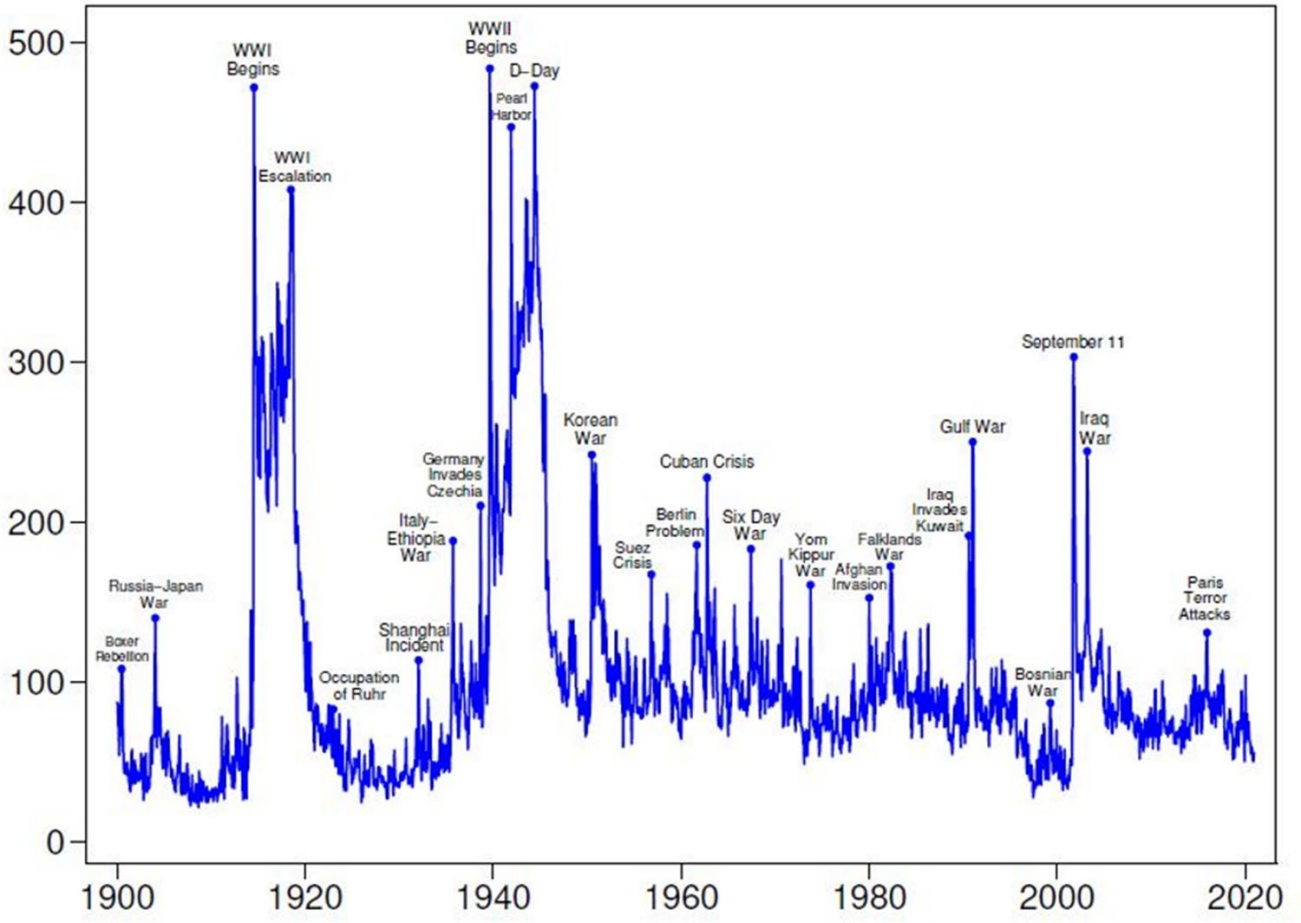
Historical GPR Index.

In extant literature, scholars have paid attention to drivers of environmental degradation, such as trade diversification^2^, energy consumption^3^, and foreign direct investment (FDI)^4^. Meanwhile, solutions for carbon neutrality have also been widely explored. These solutions include renewable energy^5^, green innovation^6^, environmental taxes^7^, and environmental policy stringency (EPS)^8^. However, some scholars contend that environmental degradation is challenging to address due to uncertainty^9,10^. In addition to economic uncertainty, geopolitical risk (GPR) stands as one of the most pervasive uncertainties worldwide. It encompasses tensions and uncertainty that arise from factors like war, peace threats, military buildups, nuclear threats, and terrorism. According to Fig. 1, since the Cuban Crisis in 1962, followed by events such as the Gulf War, September 11, and the Paris Terror Attacks, the Historical GPR Index has seen peaks. For instance, all these events significantly impact economic activities and investments. GPR has two opposite effects on the environment, which are the escalating effect and mitigating effect^11,12^. In the former, GPR reduces the use of renewable energy sources and increases the use of non-renewable ones like petroleum, which leads to higher CO_2_ emissions. Conversely, the latter effect operates in the opposite direction. In terms of theory analysis, there is no consensus on the impact of GPR on environmental quality.

**Figure 2:**
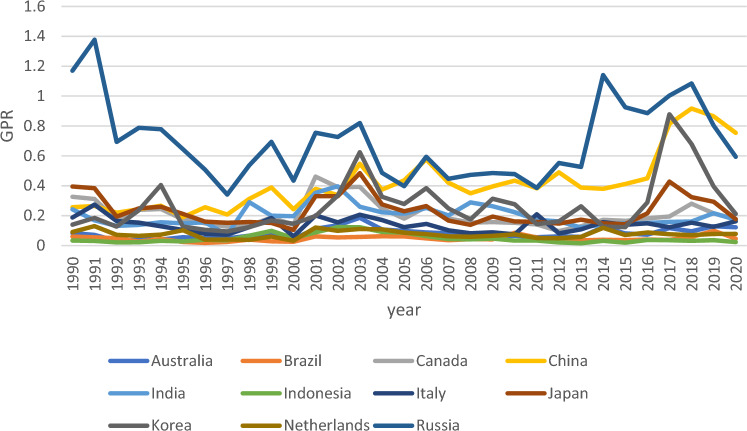
GPR index of 11 countries.

**Figure 3:**
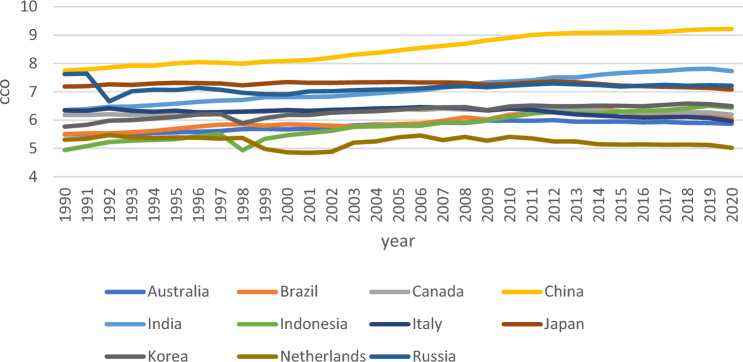
CCO_2_ emissions(log.) of 11 countries.

Despite the importance of examining how GPR influences environmental problems, little empirical research has been done on the issue. Hence, this study aims to analyse the influence of GPR on consumption-based carbon (CCO_2_) emissions. In addition, as EPS plays a crucial role in the global path towards improving environmental quality, this study incorporates the moderating effect of EPS on the above nexus. The research makes several contributions to the existing body of literature. First, the trend of GPR world has been volatile and more dynamic over the last few years. As such, GPR has garnered significant attention from numerous experts in environmental economics literature. The Ukraine/Russia conflict and the ongoing supply chain challenges that stem from the COVID-19 pandemic underscore the significance of GPR in shaping economic and environmental dynamics. Although studies have been conducted on GPR in the environmental field, environmental degradation does not entail CCO_2_. Hence, this study enriches the environmental impacts of GPR by considering CCO_2_ as the proxy of environmental degradation. Following the method of past researchers^13^, 11 economics were randomly selected from the entire research sample, which led to Figs. 2 and 3. This is to effectively illustrate how geopolitical threats and CCO_2_ emissions vary among countries. To comprehensively assess the moderating effect of EPS on the nexus between GPR and CCO_2_ emissions, data from three sources were utilised in this study: CCO_2_ from the Global Carbon Atlas, GPR from GPR index^14^, and EPS data from the OECD database. Based on data availability, a final dataset comprising 27 countries was obtained.

The use of CCO_2_ emissions provide a more comprehensive metric to account for the influence of global trade. EPS data from the Organisation for Economic Co-operation and Development (OECD) database encompasses both market-based and non-market-based policies. The GPR index was based on text analysis of adverse geopolitical events in ten prominent newspapers. The index surpasses other indicators of GPR (such as war, terrorism, and political instability) due to its incorporation of eight risk categories (e.g., war threats, peace threats, military buildups, nuclear threats, and more) and its consideration of both expected and actual GPR hazards^15,16^.

Ordinary Least Square (OLS), fixed effect model (FEM), random effect model (REM) and quantile regression were run in this study to obtain the final results. Then a robustness test was performed by replacing the independent variable and dependent variable and adding a new control variable. The sample was further divided into pre- and post-Kyoto protocol eras to look at the possible impact of outside events on the nexus.

The basic regression results indicated that GPR escalates CCO_2_. Quantile regression results indicated that GPR increases CCO_2_ emissions at all quantiles of CCO_2_ emissions while the impact showed an increasing trend before the upper quantiles. Additionally, EPS weakens the positive effect of GPR on CCO_2_ emissions. The robustness tests further confirmed these findings, supporting the robustness of the results. Specifically, a positive relationship was observed in the pre- and post-Kyoto protocol periods, even though the sign in terms of pre- Kyoto era was not significant. The impact of GPR on CCO_2_ emissions was also found to be greater in developing countries compared to developed countries Hence, this study offers a fresh analysis of the literature as well as substantial support for the use of EPS to reduce environmental pressure.

This research makes several contributions to the existing body of literature. First, this study demonstrates that GPR is a significant factor that contributes to the increase in CCO_2_ emissions. Policymakers and analysts can more effectively tackle environmental issues and promote SDG Goal 15 (Life on Land) with this perspective in mind. Second, this study documents the moderating role of EPS on the nexus between GPR and environmental quality, which has not been explored yet. While many studies^8,17^ have explored the relationship between environmental regulations and environmental quality, this research sheds light on the moderating effect of EPS on the impact of GPR on CCO_2_ emissions. This perspective provides a novel insight into the role of EPS. Third, besides traditional methods, this research uses the quantile regression model to capture the impact of GPR on environmental quality across different quantiles of CCO_2_ emissions. Hence, the results of this paper offer policymakers a fresh perspective on how to develop tailored policies.

The remainder of the paper is organised in the following parts. “Literature review” is the literature review. "Data and methodology" describes the data and methodology. "Empirical results" reports the empirical results. The last section emphasises the key conclusions.

## Literature review

### Literature on the relationship between GPR and environmental sustainability

GPR, like trade conflicts and military activities, can influence economic activity and energy use that will subsequently impact CO_2_ emissions^18^. GPR can influence CO_2_ emissions by affecting investment decisions in green technology and causing disruptions in energy supplies. Additionally, it redirects the government's focus towards managing GPR, which is an important factor that can influence the investment decision of firms^19^. For instance, investments in cleaner technologies or renewable energy can be cut due to high levels of GPR^20^, which could result in the continued reliance on fossil fuels, leading to higher CO_2_ emissions^21^. Additionally, a high level of GPR can influence governments to prioritise addressing geopolitical issues over improving environmental quality^22^. This may result in a relaxation of CO_2_ regulations, which would then lead to higher CO_2_ emissions. Finally, stability and security in one area is necessary for the access and free flow of energy resources^23^. However, GPR can disrupt energy supplies. Hence, countries may resort to using less environmentally-friendly energy sources or ramp up production in their existing fossil fuel sectors. Both scenarios have the potential to elevate CO_2_ emissions.

In terms of the empirical results regarding the relationship between GPR and environmental sustainability, scholars have held divergent views. Some scholars contend that GPR is positively correlated with CO_2_ emissions. For example, the impact of GPR on environmental quality in BRICS with continuously updated and fully modified estimators has been previously examined^22^, which were concluded to degrade environmental quality. In addition, the nexus in the same sample countries through an augmented mean group has also been studied, which found that a 1% increase in GPR leads to the increase of CO_2_ emissions by 13%^11^. Furthermore, GPR has been found to be positively correlated with CO_2_ emissions through an examination of the nexus among 25 OECD countries^24^. Notably, the heightened risks related to mineral resources was found to primarily contribute to the carbon-increase effect of GPR. GMM results have also confirmed that GPR accelerates environmental pollution in 38 developing and industrialised countries^25^. Bootstrapped ARDL was also used in one study to examine the role of GPR in sustainable environment in China^26^, which found that GPR is positively correlated with CO_2_ emissions in the long and short run.

Some studies, in contrast, have documented a negative impact. For instance, GPR has been found to be negatively correlated to environmental degradation proxied by ecological footprint consumptions in E7 economies^27^. The decline in investment and consumption activity brought by high GPR was thought to be the cause of the negative link. A similar positive relationship between GPR and environment has also been documented in the context of residential and commercial sectors in the US^28^ and France^29^. GPR has also been found to have no influence on environmental sustainability through an examination of the impact of GPR on environmental quality proxied by load capacity factor in India with ARDL method^30^.

Finally, a non-linear relationship has also been documented, where GPR increases CO_2_ emissions in countries with lower CO_2_ emissions levels and lowers them in countries with lower CO_2_ emissions levels in a sample of BRICST countries^31^. GPR depresses CO_2_ emissions in Russia and South Africa, and the effect is opposite in other BRICS countries in a study employing the non-linear autoregressive distributed lag model^32^.

Based on the inconclusive impact of GPR on environmental sustainability, this study hypothesises that GPR increases CCO_2_ emissions in the sample countries of this study (hypothesis H1).

### Literature on the relationship between EPS and environmental sustainability

EPS measures the level of stringency, which is defined as the implicit or explicit cost of environmentally harmful behaviour. This data originates from a comprehensive database that focuses primarily on policy tools that address climate change and air pollution. Hence, it is anticipated that stricter regulations can offset the negative effects of GPR on the environment. EPS is thought to have the ability to lessen the negative impacts of pollution by encouraging the development of "clean" technology and discouraging the use of "dirty" ones^33^. The mechanism through which EPS operates to reduce CO_2_ emissions is by increasing the cost of producing “dirty” products to a point where they will not be attractive^34^. A well-designed policy can assist firms in implementing eco-friendly technologies, which can result in a reduction in pollution^35^. Following this, if the benefits of regulatory compliance outweigh the expenses, it will lead to net productivity benefits, which aligns with the “narrow” version of the theory^36^. However, the expenses associated with EPS is worth noting. EPS may potentially deter investments in green innovative technologies that consequently influences environmental quality^37^. Additionally, EPS may encourage certain kinds of innovation, which can lead to net productivity loss, aligning with the 'weak' version of the theory^36^. Hence, this empirical evidence is not harmonious as to whether EPS enhances environmental quality even though EPS was found to be effective in reducing CO_2_ emissions in 20 European countries between 1995 and 2012^17^.

In the case of BRICST countries, the improvement in environmental quality has been suggested to be due to EPS^2^. Similarly, for 32 OECD countries, the emission level was found to be negatively connected with EPS^8^. However, the “green paradox”^38^ also exists, which claims that EPS may have unanticipated and undesired effects that worsen environmental degradation. GMM results have also indicated that environmental regulations have not been successful in regulating and reducing pollution as intended^39^. The increase in carbon emissions in Asia in particular has been found to be caused by environmental regulations^40^. Similarly, EPS has also been found to have little to no impact on CO_2_ reductions^41^.

Based on above discussion, the study hypothesises that EPS weakens the positive relationship between GPR and environmental sustainability (hypothesis H2).

In a nutshell, existing literature has recognised the growing importance of GPR and EPS in promoting environmental sustainability. However, current literature has not analysed the combined effect of GPR and EPS. Hence, this study addresses the first gap in the body of existing research by examining the moderating effect of EPS on the impact of GPR on environmental quality. Additionally, in the existing literature, environmental damage has often been represented by CO_2_ emissions per capita from the World Development Indicators (WDI)^11,25,31^ and ecological footprint^12,42^. However, a substantial body of research suggests that it is crucial to explore environmental conditions using alternative proxy variables, like CCO_2_ emissions. This metric, adjusted for trade effects, takes into account the role of international trade, making it a more comprehensive index of environmental degradation. Hence, this study addresses the second gap by using CCO_2_ emissions as a proxy for the environment. Furthermore, to the best of our knowledge, the analysis of the relationship between GPR and environmental quality using the panel quantile model has only been limited to BRICST countries^31^, OECD countries^24^, and GCC countries^43^. Hence, this study addresses the third gap by examining the GPR-environmental quality nexus using the quantile regression approach in 27 countries. The employment of this technique sets this study apart from those that merely rely on conventional mean-based regression models. As a result, this study enriches the existing body of literature by incorporating a novel approach that addresses individual and distributional heterogeneity.

## Data and methodology

To analyse the influence of GPR on CCO_2_ emissions, the data in this study was gathered from a variety of sources. Firstly, consumption-based carbon emissions from the Global Carbon Atlas was chosen as the dependent variable. Secondly, the GPR Index database^14^ was utilised as a measure of the GPR. Thirdly, the country-level control variables such as FDI, fiscal policy, information and communications technology (ICT), and renewable energy consumption were gathered from WDI. Finally, the EPS Index, sourced from OECD database, was used as the proxy for environment policy strength and served as the moderating variable. Country-level data from the Global Carbon Atlas, GPR Index database, and OECD database were then matched. This yielded a total of 27 countries' data spanning from 1990 to 2020, based on data availability. Additionally, the CCO_2_ emissions data for Norway was available starting from 2003. Hence, interpolation was employed to impute missing values for the control variables as practiced in past research^44^. Additionally, to mitigate the impact of outliers and large fluctuations, the data was winsorised at 1% and 99% levels.

### Dependent variable: consumption-based carbon emission

CCO_2_ emissions sourced from the Global Carbon Atlas (https://globalcarbonatlas.org/) was utilised as the dependent variable based on the approach of a previous study^5^.

### Independent variable: geopolitical risk

GPR was selected from the GPR Index database^14^. The number of articles related to unfavourable geopolitical events in each newspaper for each month across the archives of ten newspapers was counted to create this index. This index was composed of two types: Geopolitical Threats and Geopolitical Acts, based on different categories of words. This index has a number of benefits over existing indices that are currently in use, however, it has certain intrinsic drawbacks^14^. Firstly, the index covers a broader range of geopolitical events including wars, major economic crises, political conflicts, and climate change^45^. Secondly, this index also holds more reference value and timeliness as its data is derived from real-time media text-search results. These media sources collect the viewpoints of global investors, policymakers, and the public that reflect the real-time level of GPR. A higher value indicates a more unstable economic environment. The data is available at https://www.matteoiacoviello.com/gpr.htm. The simple average of the twelve months was taken to formulate a yearly index following the method of past studies^13,24,46^.

### Control variable

Previous research has indicated that the external macroeconomic environment has an impact on environmental sustainability. Foreign capital with high pollution levels has sought "pollution havens" to avoid the high costs of adhering to stringent pollution control regulations. These investors often turn to less developed nations with more lenient environmental policies, therefore, FDI enhances environmental degradation in less developed nations^47^. Next, expansionary fiscal policy involves increasing government spending, which provides the government with more funds to invest in the research and development of renewable technology and the purchase of green products^48^. Since a large number of ICT devices have high energy consumption, using ICT may result in increased CO2 emissions^49^. Also, renewable energy technology meets people’s energy demands while also mitigating pollution^50^. Hence, with reference to past research^11,48–54^, the following variables were chosen: (1) FDI, measured by foreign direct investment and net inflows (% of GDP); (2) fiscal policy, measured through the general government’s final consumption expenditure (% of GDP); (3) ICT, measured by mobile cellular subscriptions (per 100 people); and (4) renewable energy consumption (Renew), measured by percentage of total final energy consumption.

### Moderating variable: environmental policy stringency

There exists a demand for instruments to compare nations' EPS as countries implement more stringent environmental rules. This study uses the EPS index database developed by OECD based on the measurement of stringency defined as the implicit or explicit cost of environmentally harmful behaviour. This database compiles data on selected different environmental policy tools, particularly those that deal with climate change and air pollution. A smaller value indicates a less strict policy, with 0 denoting not stringent regulations.

The data for the variables above are summarised in Table 1.

**Table 1:** Variables explanations.

	Variable	Symbol	Description	Source
Dependent variable	Consumption-based CO_2_ emissions	CCO_2_	Metric tonnes of CO_2_ (log.)	(GCA, 2019 global carbon atlas) https://globalcarbonatlas.org
Independent variable	Geopolitical risk	GPR	The proxy of risk	^14^
Control variables	Foreign direct investment	FDI	Foreign direct investment, net inflows (% of GDP)	WDI
	Fiscal policy	Fiscal	General government final consumption expenditure (% of GDP)	WDI
	ICT	ICT	Mobile cellular subscriptions (per 100 people)	WDI
	Renewable energy consumption	Renew	Renewable energy consumption (% of total final energy consumption)	WDI
Moderating variable	environmental policy stringency	EPS	the proxy of environmental law	OECD database

### Econometric model

This study employs OLS, FEM, and REM to thoroughly examine the relationship between GPR and CCO_2_ emissions. The Breusch Pagan and Lagrangian Multiplier (BP and LM) test was the foremost step as this test can detect whether pooled or panel data is optimal. If the p-value of BP test and chi-square of LM test is significant at level 5%, then panel data is chosen. Both FEM and REM are employed in this study to deal with the panel data. The Hausman test is used to choose the model suitable for this research based on the null hypothesis. The FEM is chosen to analyse the data if the null hypothesis is rejected (or when the prob. < 0.05).

H0: the random effect is appropriate

H1: the random effect is not appropriate

Quantile regression was utilised to get a comprehensive result. This method is preferred by scholars^55,56^ over mean-based estimation techniques like OLS for the following reasons. Firstly, it can yield robust results even when the data exhibits heavy tails. Secondly, this statistical approach examines the influence of GPR on CCO_2_ emissions across various quantiles, as illustrated in Eq. (2). Consequently, it can explain how GPR affects CCO_2_ emissions at relatively lower, middle, and upper levels.

The following empirical equation was thus proposed:

1 $$CCO2it{ = }{\upalpha 0} + {\upalpha 1}GPRit + \sum\limits_{a = 1}^{4} {\upbeta a} CCit +\upvarepsilon it$$

2 $$Q{\rm T}(CCO2it\left| {} \right. GPRitCCit) = \alpha 0{\rm T} + \alpha 1{\rm T}GPRit + \sum\limits_{a = 1}^{4} {\beta a{\rm T}} CCit + \varepsilon it$$

3 $$CCO2it{ = }{\upalpha 0} + {\upalpha 1}GPRit + {\upalpha 2}EPSit + {\upalpha 3}GPRit*EPSit + \sum\limits_{a = 1}^{4} {\upbeta a} CCit +\upvarepsilon it$$

where: CCO_2it_ is the log term of CCO_2_ emissions of country i at time t, GPR_it_ is the level of geopolitical risk of country i at time t, EPS_it_ is the environmental policy stringency of country i at time t, CC_it_ is the control variable of country i at time t, ƹ_ijt_ is the error term, Q_T_ is the conditional quantile, _T_ represents the quantile.

## Empirical results

### Descriptive statistics

Table 2 presents the descriptive statistics of variables for all countries in the sample.

**Table 2:** Descriptive statistics for all countries.

Variable	N	Mean	sd	Min	Max
CCO	824	5.88	1.24	3.75	9.21
GPR	837	0.28	0.49	0.01	4.35
FDI	837	3.34	6.45	-7.42	43.49
Fiscal	837	18.41	4.44	5.69	27.93
ICT	837	69.10	50.82	0.00	172.20
Renew	837	18.20	16.08	0.44	61.37
EPS	837	1.94	1.18	0.00	4.89

In terms of the dependent variable, the mean value of CCO_2_ emissions was 5.88 with a standard deviation of 1.24. In the sample, the independent variable's mean value was 0.28. In the meantime, there was a significant range in both the maximum and minimum values of GPR, which reflects the diversity in GPR among nations. Significant fluctuations around the sample mean can also be observed for other control variables.

Table 3 presents Pearson’s correlation matrix that displays coefficients between variables. Concerning the dependent variable, a negative correlation was found between CCO_2_ emissions and FDI, Fiscal, ICT, EPS, and Renew. Conversely, there was a positive correlation between CCO_2_ emissions and GPR. Additionally, the maximum correlation between the explanatory variables was found to be lower than 0.8, demonstrating that the regression estimation was not multicollinear. In a multivariate study, multicollinearity exists if the correlation coefficients with the explanatory variables are more than 0.8.

**Table 3:** Correlation matrix.

	CCO	GPR	FDI	Fiscal	ICT	Renew	EPS
CCO	1						
GPR	0.604***	1					
FDI	− 0.168***	− 0.092***	1				
Fiscal	− 0.394***	− 0.139***	0.187***	1			
ICT	− 0.087**	− 0.017	0.131***	0.241***	1		
Renew	− 0.323***	− 0.289***	− 0.121***	− 0.052	− 0.019	1	
EPS	− 0.193***	− 0.036	0.091***	0.368***	0.726***	− 0.014	1

*p < 0.1, **p < 0.05, ***p < 0.01.

The VIF value in Table 4 indicates that there was no significant multicollinearity among the variables in the regression model provided the maximum VIF value is 2.31.

**Table 4:** VIF.

Variable	VIF	1/VIF
EPS	2.31	0.432
ICT	2.14	0.468
Fiscal	1.23	0.813
GPR	1.13	0.882
Renew	1.13	0.886
FDI	1.07	0.932
Mean/VIF	1.5	

The Pesaran CD^57^, Pesaran scaled LM, and Breusch–Pagan LM tests^58^ were used to test the cross-sectional dependence of data. Based on the results, there was no cross-sectional dependence, which confirmed the null hypothesis. All results demonstrated in Table 5 were statistically significant at the 1% level, suggesting a significant interdependence and cross-sectional correlation among the variables. The test's overall finding was that all variables were cross-sectionally dependent.

**Table 5:** Results of cross-sectional dependence tests.

Test	Statistics
Pesaran CD test	2.473**
Pesaran scaled LM test	2.526**
Breusch–Pagan LM test	8765.06***

*p < 0.1, **p < 0.05, ***p < 0.01.

To explore the integration order of separate variables, the LLC^59^, IPS^60^, HT^61^, ADF-Fisher^62^, and PP-Fisher^63^ tests were performed, and it was found that at the level form, not all variables were stationary, but when they reached their first difference, as shown in Table 6 they became stationary.

**Table 6:** Stationarity test results.

	Variables	CCO	griska	FDI	Fiscal	ICT	Renew	EPS
Level form	LLC	–	− 6.568***	− 8.055***	− 2.201**	− 2.354***	1.207	− 0.402
	IPS	− 4.679***	− 8.710***	− 10.524***	− 2.539***	3.205	− 2.224**	− 3.591***
	HT	–	− 7.846***	− 13.556***	− 1.493*	5.479	− 1.037	0.770
	DF	124.614***	115.621***	161.108***	76.800**	35.269	44.099	56.875
	PP	92.845***	127.288***	246.851***	68.686*	8.635	66.258	74.177**
1st	LLC	–	− 8.059***	− 17.042***	− 6.980***	− 5.380***	− 7.304***	− 11.014***
	IPS	− 16.454***	− 18.193***	− 17.672***	− 13.498***	− 9.081***	− 15.518***	− 16.052***
	HT	–	− 34.747***	− 37.631***	− 23.652***	− 13.665***	− 24.473***	− 28.742***
	DF	375.489***	300.040***	587.072***	279.106***	129.924***	309.967***	341.171***
	PP	771.090***	909.330***	921.004***	444.839***	204.869***	681.162***	651.615***

***Indicates a 1% significance level. The Stata commands xtunitroot llc (for LLC), xtunitroot ips (for IPS), xtunitroot ht for (for HT), xtunitroot fisher dfuller (for ADF-Fisher), and xtunitroot fisher pperron (for PP-Fisher) were employed to estimate the results in this table. LLC and HT were not able to be performed in terms of CCO_2_ emissions as strongly balanced data was needed.

### Basic results

In Table 7, GPR and control variables were included in the model. In the preliminary stage, the results from Table 7 show that the fixed effect model was most suitable to be used in this study as the p value of BP test and Chi-square of LM test was significant at 1% level or lower and the p value of the Hausman test was significant at 1% level or lower.

**Table 7:** Basic regression result.

	ols	fe	re
GPR	1.213*** (0.06)	0.067*** (0.02)	0.075*** (0.02)
FDI	− 0.017*** (0.00)	− 0.001 (0.00)	− 0.001 (0.00)
Fiscal	− 0.092*** (0.01)	0.015*** (0.00)	0.013*** (0.00)
ICT	0.000 (0.00)	0.002*** (0.00)	0.002*** (0.00)
Renew	− 0.018*** (0.00)	− 0.033*** (0.00)	− 0.033*** (0.00)
constant	7.576*** (0.14)	6.036*** (0.07)	6.054*** (0.16)
N	824	824	824
r2	0.510	0.685	
r2_a	0.507	0.672	
F	170.260***	343.829***	
BP LM	chibar2(01) = 8928.26***		
chi2			1629.747***
Hausman		chi2(5) = 49.73***	

Standard errors in parentheses.

*p < 0.1, **p < 0.05, ***p < 0.01.

The FEM in Table 7 shows that GPR has a significant and positive effect on CCO_2_ emissions. A 1% increase in GPR leads to an increase in CCO_2_ emissions by 0.067%. The regression results indicate that GPR negatively impacts environmental sustainability in the sample countries. This confirms hypothesis H1. The above effect can be explained by the following reasons. Firstly, as the level of GPR increases, the risk premium of an investment rises. This means that investors may postpone or even reconsider the viability of the investment. For the private sector, there may also be concerns about the returns on investments in environmentally-friendly technologies and projects. In the case of the public sector, this can lead to a decrease in the investments in green technology, with the tendency to prioritise short-term financial gains over long-term sustainability projects. In addition, GPR can impact the sustainability of the international carbon reduction process. For example, when it comes to environmental challenges, tensions between countries can make international cooperation difficult. Cooperative efforts to combat climate change, preserve ecosystems, and exchange sustainable technologies may also face delays or encounter obstacles due to geopolitical conflicts. Additionally, geopolitical tensions have led nations to prioritise energy independence. Instead of relying on environmentally-friendly but highly import-dependent products, some nations may opt for more accessible yet carbon-intensive energy sources, potentially leading to an increase in carbon emissions. Finally, geopolitical disputes make it challenging to implement and maintain consistent environmental regulations due to political instability or weak governance^24^. This finding is consistent with a previous study^24^ on the relationship between GPR and CO_2_ emissions within the context of the OECD countries. It also aligns with another study^11^ which confirmed that GPR escalates CO_2_ emission in BRICS economics. Similar findings were also reported for BRICST countries^31^ and for the transportation sector in the US^28^.

Regarding the control variables, the regression results showed that renewable energy and FDI negatively contributed to CCO_2_ emissions, while fiscal policy and ICT had a positive impact on CCO_2_ emissions.

The results suggest a negative association between renewable energy and CCO_2_ emissions. Renewable energy such as wind, solar, and hydroelectric can generate electricity without emitting any pollutants into the atmosphere^64^. It is worth noting that the findings confirm the conclusions in the works of several past research works^65,66^. Regarding the role of FDI, the regression results indicate that FDI can alleviate environmental pressure. This phenomenon can be attributed to the pollution halo hypothesis. According to this hypothesis, FDI brings advanced technologies and green practices from developed countries to the developing country, enabling these nations to produce in a more environmentally-friendly manner, which may be contradictory^67^. In terms of fiscal policy, the positive coefficient of fiscal policy indicates that fiscal policy, which increases government spending^68^, aggravates CCO_2_ emissions. Hence, this will greatly stimulate overall economic demand, thereby increasing CCO_2_ emissions which is in line with the findings of previous researchers^69^. In addition, the use of ICT may contribute to increased CCO_2_ emissions due to the high energy consumption associated with the large number of ICT devices^49^.

### Quantile regression

**Figure 4:**
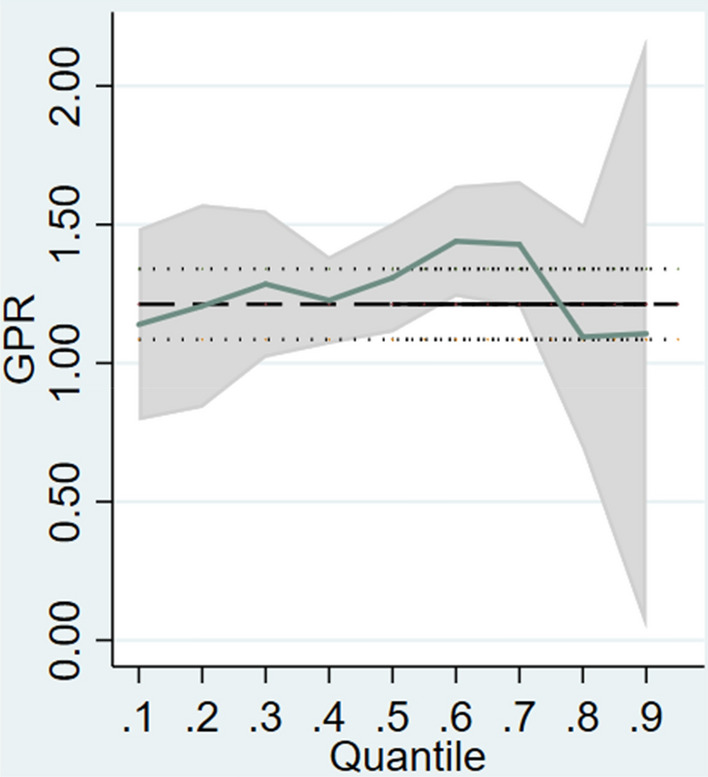
Graphical evaluation of coefficients of quantile regression.

To attain a more robust result, the model was run by the panel quantile regression which offers a more thorough study for model estimation at multiple quantiles^70^. This method has two advantages. First, quantile regression is considered more reliable when the data is not normally distributed^71^. Secondly, quantile regression is a useful tool for estimating the significant impact of extreme values^24^. While the traditional econometric model provides the average effect of the independent variable, panel quantile regression not only provides results at different quantile and complies with the non-normality requirements, but also solves issues with variable slope coefficients and cross-sectional dependence^72^. This technique is widely used in the field of environmental economics. Hence, three kinds of quantiles were chosen based on past research^73^, namely the lower (10th–30th), middle (40th–60th), upper quantiles (70th–90th) as depicted in Table 8 and Fig. 4. The magnitude supports the application of quantile regression because the impact of GPR on CCO_2_ emissions is heterogeneous across quantiles.

**Table 8:** Quantile regression.

	Lower			Middle			Upper		
	0.1	0.2	0.3	0.4	0.5	0.6	0.7	0.8	0.9
GPR	1.139***	1.206***	1.285***	1.227***	1.308***	1.440***	1.428***	1.095***	1.107***
	(0.11)	(0.09)	(0.08)	(0.07)	(0.06)	(0.09)	(0.11)	(0.09)	(0.15)
FDI	− 0.007	− 0.019***	− 0.020***	− 0.015***	− 0.011**	− 0.011	− 0.012	− 0.017**	− 0.016
	(0.01)	(0.01)	(0.01)	(0.01)	(0.00)	(0.01)	(0.01)	(0.01)	(0.01)
Fiscal	− 0.046***	− 0.079***	− 0.076***	− 0.080***	− 0.084***	− 0.093***	− 0.099***	− 0.135***	− 0.162***
	(0.01)	(0.01)	(0.01)	(0.01)	(0.01)	(0.01)	(0.01)	(0.01)	(0.02)
ICT	0.001	0.002**	0.001	0.001**	0.002***	0.001	− 0.000	0.001	0.001
	(0.00)	(0.00)	(0.00)	(0.00)	(0.00)	(0.00)	(0.00)	(0.00)	(0.00)
Renew	− 0.018***	− 0.025***	− 0.025***	− 0.023***	− 0.022***	− 0.019***	− 0.010***	− 0.012***	− 0.012***
	(0.00)	(0.00)	(0.00)	(0.00)	(0.00)	(0.00)	(0.00)	(0.00)	(0.00)
constant	5.476***	6.701***	6.939***	7.156***	7.266***	7.602***	7.963***	8.991***	9.845***
	(0.26)	(0.19)	(0.18)	(0.15)	(0.13)	(0.21)	(0.24)	(0.21)	(0.33)
N	824	824	824	824	824	824	824	824	824

Standard errors in parentheses.

*p < 0.1, **p < 0.05, ***p < 0.01.

The coefficients of GPR were found to be positive and significant across the distribution. As we transition from lower quantiles to middle quantiles, the magnitude also increases. Initially, a 1% rise in GPR leads to a 1.139% increase in CCO_2_ emissions at the 10th percentile of CCO_2_ emissions. However, the elasticity surges to 1.440% at the 60th percentile. In contrast, the magnitude decreases in upper quantiles, yet remains positive and statistically significant, which contradict findings from past research^31,32^ which proposed that GPR deflates the environmental quality at lower quartiles while the effect is reverse at other quantiles. GPR has also been contradictorily been documented^32^ to depress CO_2_ emissions in Russia and South Africa and escalate emissions in other BRICS countries with the method of non-linear autoregressive distributed lag model. The varying positive association between GPR and CCO_2_ emissions confirms hypothesis H1. This variation suggests that the impact of GPR is contingent upon the degree of environmental degradation. The increasing trend before the upper quantile can be explained by the following. First, in countries with higher CCO_2_ emissions quantiles, technology is usually less advanced, which creates a gap between countries in higher and lower CCO_2_ emissions quantiles. A wider technology gap will also result in a country holding a lower position in the global value chain and engage in less environmentally-friendly production^74^. The lower global value chain position also makes these countries more susceptible to disruptions caused by geopolitical events. Secondly, countries with higher CCO_2_ emissions quantiles may have more resource-intensive industries and are more likely to be influenced by GPR that relate to resource and trade. Firms also tend to resort to polluting production methods due to concerns on GPR^11^. The diminishing effect in upper quantiles can be explained by market pressures and environment policy constrains. In terms of the former, industries in countries with higher CCO_2_ emissions quantiles may experience pressure to adopt cleaner practices due to consumer and market demands for sustainability and environmental responsibility. In contrast, stringent environmental regulations are implemented by the government due to severe environmental pollution. This, to some extent, counteracts the dependence on polluting production methods and heavily-polluting energy sources caused by GPR.

In conclusion, as we progress from lower to higher quantiles, the coefficients exhibit an increasing trend. This suggests that GPR has a particularly notable impact in countries with higher CCO_2_ emissions levels.

### Moderating effect of EPS

Different exogenous shocks, such as global economic uncertainty and EPS, may impact the way GPR affects CCO_2_ emissions. Various levels of EPS may produce different results. For instance, when EPS increases, the profit from using the polluting producing method and polluting energy may be halted or reduced. In line with the basic regression findings, the coefficient of GPR was found to be significantly positive at the 1% level. Furthermore, the interaction term (GPR* EPS) exhibited a significant negative coefficient, as demonstrated in Table 9. This signifies that the negative effects of GPR on environmental sustainability could be somewhat mitigated by an increase in EPS level. Hence, the negative coefficient confirms hypothesis H2. This can be explained by regulatory compliance. EPS backed by robust enforcement mechanisms can elevate the cost of polluting production and the use of non-clean energy sources, resulting in a mitigation effect on CCO_2_ emissions. Furthermore, stringent regulations impose limits on emissions and encourage the adoption of greener practices and technologies. A similar carbon reduction effect of EPS has been documented^75^ when analysing the moderating effect of EPS on the impact of financial development on environmental quality.

**Table 9:** Moderating effect.

	ols	fe	re
GPR	0.801*** (0.13)	0.118*** (0.03)	0.124*** (0.03)
EPS	− 0.191*** (0.04)	0.081*** (0.01)	0.080*** (0.01)
Interact	0.251*** (0.07)	− 0.041*** (0.01)	− 0.040*** (0.01)
FDI	− 0.017*** (0.00)	− 0.000 (0.00)	− 0.000 (0.00)
Fiscal	− 0.082*** (0.01)	0.010*** (0.00)	0.008** (0.00)
ICT	0.003*** (0.00)	0.001*** (0.00)	0.001*** (0.00)
Renew	− 0.017*** (0.00)	− 0.035*** (0.00)	− 0.035*** (0.00)
constant	7.596*** (0.14)	6.078*** (0.06)	6.092*** (0.16)
N	824	824	824
r2	0.524	0.710	
r2_a	0.520	0.698	
F	128.513***	276.802***	
BP LM	chibar2(01) = 8704.77***		
chi2			1843.699***
Hausman		chi2(7) = 47.47***	

Standard errors in parentheses.

*p < 0.1, **p < 0.05, ***p < 0.01.

### Robust test

In this section, four robustness tests were conducted to validate the basic findings. Initially, the independent variable was replaced. Then, the control variable was added to re-examine the underlying link. In addition, the dependent variable was replaced to assess the robustness of the basic regression model. Finally, the sample was segmented into pre- and post-Kyoto protocol periods to investigate the potential influence of external events on the nexus.

#### Robust test1: replacing independent variable

Following a method done in past literature^13^, the annual GPR index calculated by the geometric mean method (GPR-G) was generated to test the robustness of the test. The results in Table 10 show that the main findings still hold.

**Table 10:** Robust test1: replace independent variable.

	ols	fe	re
GPR-G	1.330*** (0.07)	0.090*** (0.03)	0.101*** (0.03)
FDI	− 0.016*** (0.00)	− 0.001 (0.00)	− 0.001 (0.00)
Fiscal	− 0.092*** (0.01)	0.014*** (0.00)	0.012*** (0.00)
ICT	0.000 (0.00)	0.002*** (0.00)	0.002*** (0.00)
Renew	− 0.017*** (0.00)	− 0.033*** (0.00)	− 0.033*** (0.00)
constant	7.559*** (0.14)	6.036*** (0.07)	6.053*** (0.16)
N	824	824	824
r2	0.520	0.686	
r2_a	0.517	0.673	
F	177.240***	345.341***	
BP LM	chibar2(01) = 9149.93***		
chi2			1638.257***
Hausman		chi2(5) = 49.39***	

Standard errors in parentheses.

*p < 0.1, **p < 0.05, ***p < 0.01.

#### Robust test2: add extra control variable

The absence of relevant variables was likely to decrease the validity of empirical findings and bring about estimation basis. Therefore, a factor was added to the model to see if the main findings change. FD (financial development) facilitates green projects in obtaining loans, thereby reducing CO_2_ emissions^49^. Hence, the variable of FD was incorporated into the analysis following the method of past studies^49,76^. FD is proxied by domestic credit to private sector’s percentage of GDP. Subsequently, this variable was introduced into the regression model. The corresponding outcomes are reported in Table 11. The regression outcomes validated the robustness of the fundamental regression analysis.

**Table 11:** Robust test2: adding control variable FD.

	ols	fe	re
GPR	1.190*** (0.07)	0.059*** (0.02)	0.066*** (0.02)
FDI	− 0.016*** (0.00)	0.000 (0.00)	− 0.000 (0.00)
Fiscal	− 0.093*** (0.01)	0.012*** (0.00)	0.011*** (0.00)
ICT	0.000 (0.00)	0.001*** (0.00)	0.001*** (0.00)
Renew	− 0.018*** (0.00)	− 0.034*** (0.00)	− 0.034*** (0.00)
FD	0.001 (0.00)	0.002*** (0.00)	0.002*** (0.00)
constant	7.531*** (0.15)	5.961*** (0.06)	5.975*** (0.16)
N	824	824	824
r2	0.511	0.713	
r2_a	0.507	0.702	
F	142.234***	327.863***	
BP LM	chibar2(01) = 8969.87***		
chi2			1872.294***
Hausman		chi2(6) = 47.51***	

Standard errors in parentheses.

*p < 0.1, **p < 0.05, ***p < 0.01.

#### Robust test3: replace dependent variable

The model was re-estimated by replacing the dependent variable with CO_2_ emissions metric tons per capita(log.) (CE). In line with past studies^49,77–81^, CE was chosen as one indicator of the quality of the environment. The results are reported in Table 12. The regression results showed that the basic regression remains robust.

**Table 12:** Robust test3: replace dependent variable.

	CE		
	ols	fe	re
GPR	0.280*** (0.03)	0.067*** (0.02)	0.068*** (0.02)
FDI	− 0.006** (0.00)	− 0.000 (0.00)	− 0.000 (0.00)
Fiscal	0.065*** (0.00)	− 0.000 (0.00)	0.001 (0.00)
ICT	0.001** (0.00)	0.001*** (0.00)	0.001*** (0.00)
Renew	− 0.023*** (0.00)	− 0.033*** (0.00)	− 0.033*** (0.00)
constant	0.991*** (0.08)	2.420*** (0.05)	2.400*** (0.10)
N	837	837	837
r2	0.558	0.759	
r2_a	0.555	0.750	
F	209.462***	508.259***	
BP LM	chibar2(01) = 9975.67***		
chi2			2522.288***
Hausman		chi2(5) = 17.15***	

Standard errors in parentheses.

*p < 0.1, **p < 0.05, ***p < 0.01.

#### Robust test4: the impact of the Kyoto protocol’s enforcement

All sample countries in this study were signatories to the Kyoto Protocol and were therefore influenced by it. In 2005, the Kyoto Protocol officially entered into force after being agreed upon in 2003. Therefore, the pre-Kyoto era was firstly examined, and unexpectedly, it was found that the GPR’s sign was positive but not significant, indicating that earlier GPRs were wholly ineffective in escalating CO_2_ emissions. However, when the post-Kyoto era was examined, similar signs were observed to the basic regression model as shown in Table 13, which confirmed the basic regression results.

**Table 13:** Robust test4: The impact of the Kyoto protocol’s enforcement.

	Pre-Kyoto			Post-Kyoto		
	ols	fe	re	ols	fe	re
GPR	1.134*** (0.08)	0.031 (0.02)	0.039* (0.02)	1.312*** (0.10)	0.094** (0.04)	0.116** (0.05)
FDI	− 0.015 (0.01)	0.000 (0.00)	− 0.000 (0.00)	− 0.020*** (0.01)	0.001 (0.00)	0.001 (0.00)
Fiscal	− 0.068*** (0.01)	0.026*** (0.01)	0.023*** (0.01)	− 0.086*** (0.01)	0.006 (0.01)	0.004 (0.01)
ICT	− 0.005*** (0.00)	0.001*** (0.00)	0.001*** (0.00)	− 0.009*** (0.00)	0.002*** (0.00)	0.002*** (0.00)
Renew	− 0.011*** (0.00)	− 0.036*** (0.00)	− 0.034*** (0.00)	− 0.028*** (0.00)	− 0.032*** (0.00)	− 0.032*** (0.00)
constant	7.061*** (0.21)	5.889*** (0.10)	5.900*** (0.19)	8.772*** (0.23)	6.159*** (0.09)	6.199*** (0.18)
N	392	392	392	432	432	432
r2	0.477	0.433		0.617	0.603	
r2_a	0.470	0.384		0.612	0.572	
F	70.470***	55.042***		137.232***	121.582***	
BP LM	chibar2(01) = 1860.10***			chibar2(01) = 2318.16***		
chi2			243.780***			575.655***
Hausman		chi2(5) = 43.48***			chi2(5) = 35.66***	

Standard errors in parentheses.

*p < 0.1, **p < 0.05, ***p < 0.01.

### Heterogeneity test

Based on the classification of United Nations (https://unstats.un.org/unsd/methodology/m49/historical-classification-of-developed-and-developing-regions.xlsx), this study divided the sample country into developed and developing countries as demonstrated in Table 14 and reperformed the regression model. For developing country samples, the REM was the most suitable while the FEM was the most suitable for developed countries according to Table 15. The impact of GPR on environmental sustainability was found to be smaller in developed countries compared to developing countries. This may be because developed countries often have more advanced infrastructure and technology to support renewable energy development^82^. A well-established domestic renewable energy system enables a relatively independent energy supply. When facing GPRs, countries are less likely to heavily consume fuel energy. This results in smaller coefficients regarding developed countries.

**Table 14:** Classification.

Country	Type	Country	Type	Country	Type	Country	Type
Brazil	Developing	Australia	Developed	Hungary	Developed	Russia	Developed
China	Developing	Belgium	Developed	Italy	Developed	Spain	Developed
India	Developing	Canada	Developed	Japan	Developed	Sweden	Developed
Indonesia	Developing	Denmark	Developed	Netherlands	Developed	Switzerland	Developed
Korea	Developing	Finland	Developed	Norway	Developed	United Kingdom	Developed
South Africa	Developing	France	Developed	Poland	Developed	United States	Developed
Turkey	Developing	Germany	Developed	Portugal	Developed

**Table 15:** Robust test4.

	Developed			Developing		
	ols	fe	re	ols	fe	re
GPR	1.130*** (0.05)	0.036* (0.02)	0.047** (0.02)	3.935*** (0.29)	0.416*** (0.09)	0.425*** (0.09)
FDI	− 0.026*** (0.00)	0.000 (0.00)	− 0.000 (0.00)	0.285*** (0.03)	0.015* (0.01)	0.016* (0.01)
Fiscal	− 0.042*** (0.01)	0.010*** (0.00)	0.009** (0.00)	− 0.028** (0.01)	0.011 (0.01)	0.010 (0.01)
ICT	0.003*** (0.00)	0.001*** (0.00)	0.001*** (0.00)	0.002** (0.00)	0.003*** (0.00)	0.003*** (0.00)
Renew	− 0.042*** (0.00)	− 0.026*** (0.00)	− 0.026*** (0.00)	0.007** (0.00)	− 0.032*** (0.00)	− 0.032*** (0.00)
constant	6.694*** (0.18)	5.742*** (0.07)	5.752*** (0.16)	5.335*** (0.22)	6.860*** (0.13)	6.859*** (0.36)
N	607	607	607	217	217	217
r2	0.689	0.395		0.595	0.861	
r2_a	0.686	0.370		0.585	0.854	
F	266.001***	75.872***		62.010***	254.311***	
BP LM	chibar2(01) = 5549.82***			chibar2(01) = 756.79***		
chi2			376.316***			1242.266***
Hausman		chi2(5) = 43.24***			chi2(5) = 9.37*	

Standard errors in parentheses.

*p < 0.1, **p < 0.05, ***p < 0.01.

## Conclusion and policy recommendations

### Conclusion

In the current modern era, GPR is a significant issue that has a strong environmental impact and economic impact on nations. Hence, this paper studies the impact of GPR on CCO_2_ emissions based on the data collected from 27 countries ranging from 1990 to 2020. First, the GPR-CCO_2_ emissions nexus was tested and then checked with regards to the heterogeneous impact of GPR at different quantiles of CCO_2_ emissions. Furthermore, this study examined the moderating effect of EPS on the above nexus. Several robustness tests were used to check the basic results. Finally, a heterogeneity test was performed in developing countries and developed countries.

The results of the study suggest that GPR can significantly increase CCO_2_ emissions and has a greater and more substantial impact on higher quantiles of CCO_2_ emissions. Meanwhile, EPS can negatively moderate the nexus among GPR and CCO_2_ emissions. In other words, the negative effects of GPS on environmental quality could be somewhat offset by improvements in EPS level. The robust tests confirm the basic regression results. Additionally, upon dividing the sample period into pre- and post-Kyoto periods, it was observed that the impact of GPR aligns with the basic regression model during the post-Kyoto periods. However, it is interesting to note that the effect of GPR in the pre- Kyoto period is positive but insignificant. The heterogeneity test indicates that the impact of GPR on CCO_2_ emissions is greater in developing countries compared in developed countries.

### Policy recommendations

This research has several implications for policy. First, the relationship between GPR and a country's environmental deregulation suggests that higher GPR may be detrimental to efforts aimed at improving environmental sustainability. To address this issue, the government should provide a stable political environment and a sound legal system, thereby attracting more investors to participate in environmental projects. Besides that, energy transition could be promoted by adopting policies to reduce reliance on high-carbon-emission energy sources and instead use cleaner and renewable energy sources. Finally, the government can strengthen environmental regulation to ensure the enforcement of environmental laws and enhance the effectiveness of environmental protection measures.

Second, the heterogeneous impact of GPR at different quantiles serve as a reminder that low- and middle-emissions countries should pay closer attention to reduce CCO_2_ emissions, as the influence of GPR becomes stronger in higher level of CCO_2_ emissions countries. Besides that, every country has different circumstances, thus, policies must be developed, put into practice, and be continuously improved to reflect those particulars.

Third, the government needs to make every effort to optimise the benefits of EPS. EPS weakens the negative impact of GPR by enforcing regulatory compliance and promoting green production. As a result, it may be more efficient to put strict measures into place, such extending the scope of environmental taxes and imposing different tax rates according to the degree of environmental harm. However, softer methods can also prove to be highly effective. It is imperative to make proactive investments in clean technology research and development. These technologies directly support source reduction of emissions by providing workable substitutes for high-emission processes. Promoting garbage recycling and sustainable consumption can also greatly increase public participation in the battle against environmental deterioration. By implementing these strategies, policymakers will be able to successfully manage the complex relationship between GPR, environmental quality, and EPS.

### Limitations

First, this study’s sample is constrained by data availability. It includes countries where the independent, dependent, and control variables intersect. In addition, data for only 27 countries and spanning from 1990 were used for this study.

Second, the empirical model only employed the techniques of OLS, FE, RE, and panel quantile regression.

### Supplementary Information

Supplementary Information.

## Data Availability

Data is provided within supplementary information files.
